# Supplementary figures and images for: S1P-S1PR1 signaling impairs CD8+ T cell metabolism and effector function in tumors
Source: EMBO Rep. 2026 Mar 19;27(8):2000–28. doi: 10.1038/s44319-026-00734-3 (PMC13121723; doi:10.1038/s44319-026-00734-3)

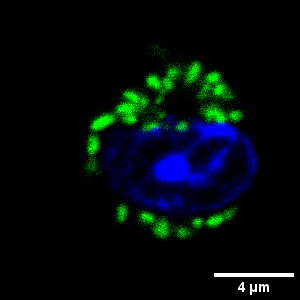

Supplement: Supplementary file 9 — Figure EV2 Source Data [file 44319_2026_734_MOESM9_ESM.zip › EV2/2B/2B/4857 MN-1.tif]

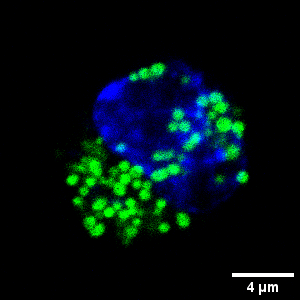

Supplement: Supplementary file 9 — Figure EV2 Source Data [file 44319_2026_734_MOESM9_ESM.zip › EV2/2B/2B/4869 MN-1.tif]

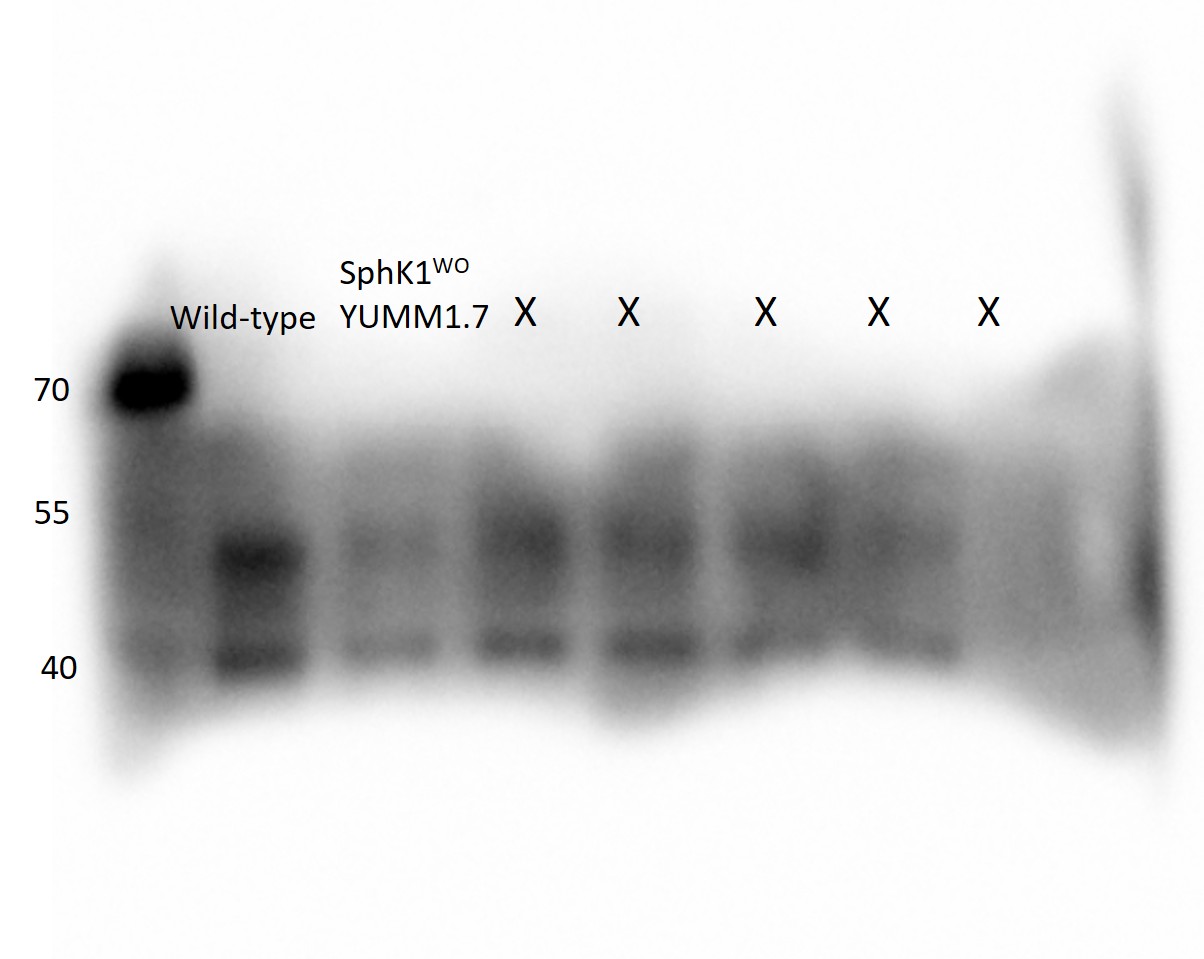

Supplement: Supplementary file 10 — Figure EV3 Source Data [file 44319_2026_734_MOESM10_ESM.zip › EV3/3A/Sphk1 new.jpg]

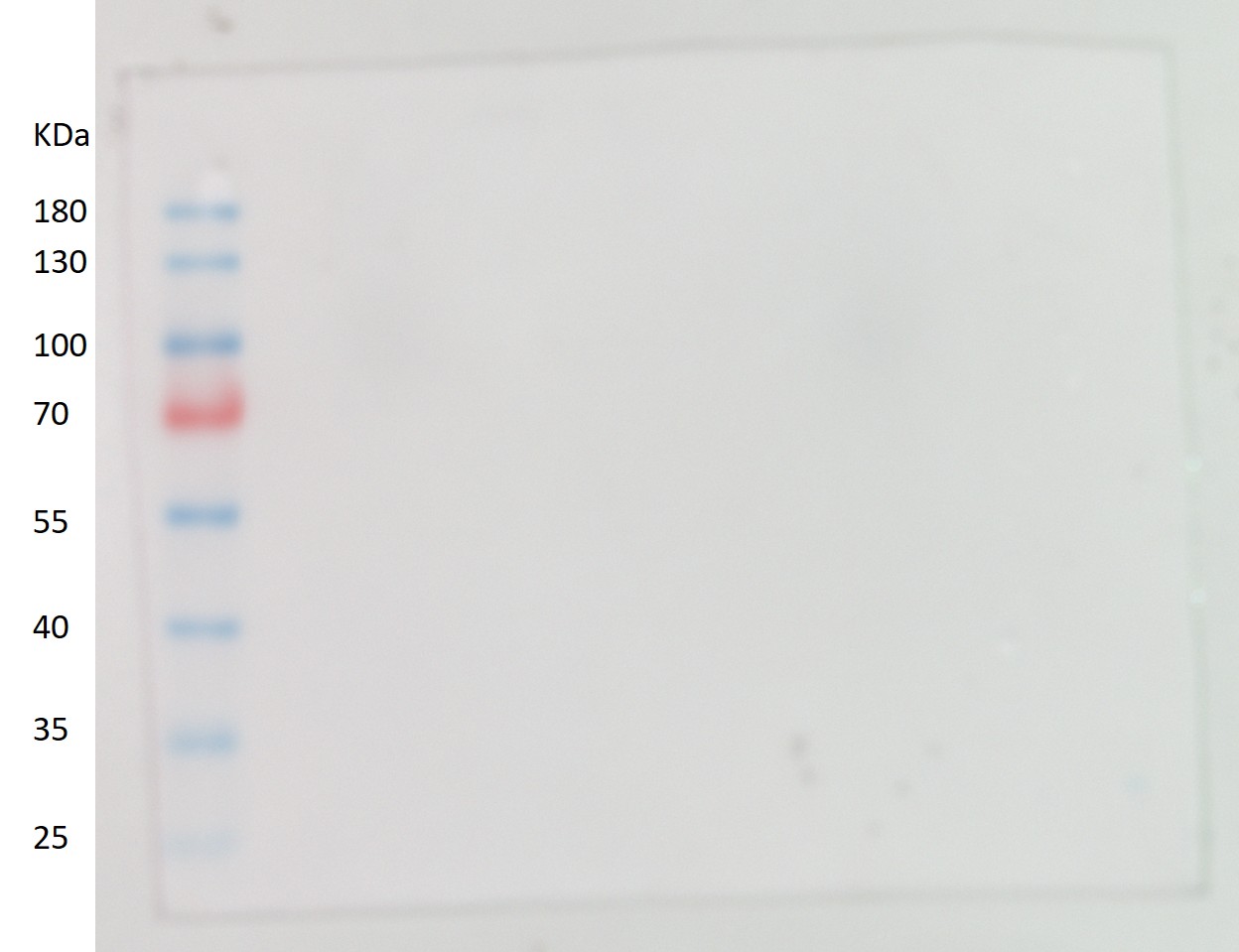

Supplement: Supplementary file 10 — Figure EV3 Source Data [file 44319_2026_734_MOESM10_ESM.zip › EV3/3A/Ladder .jpg]

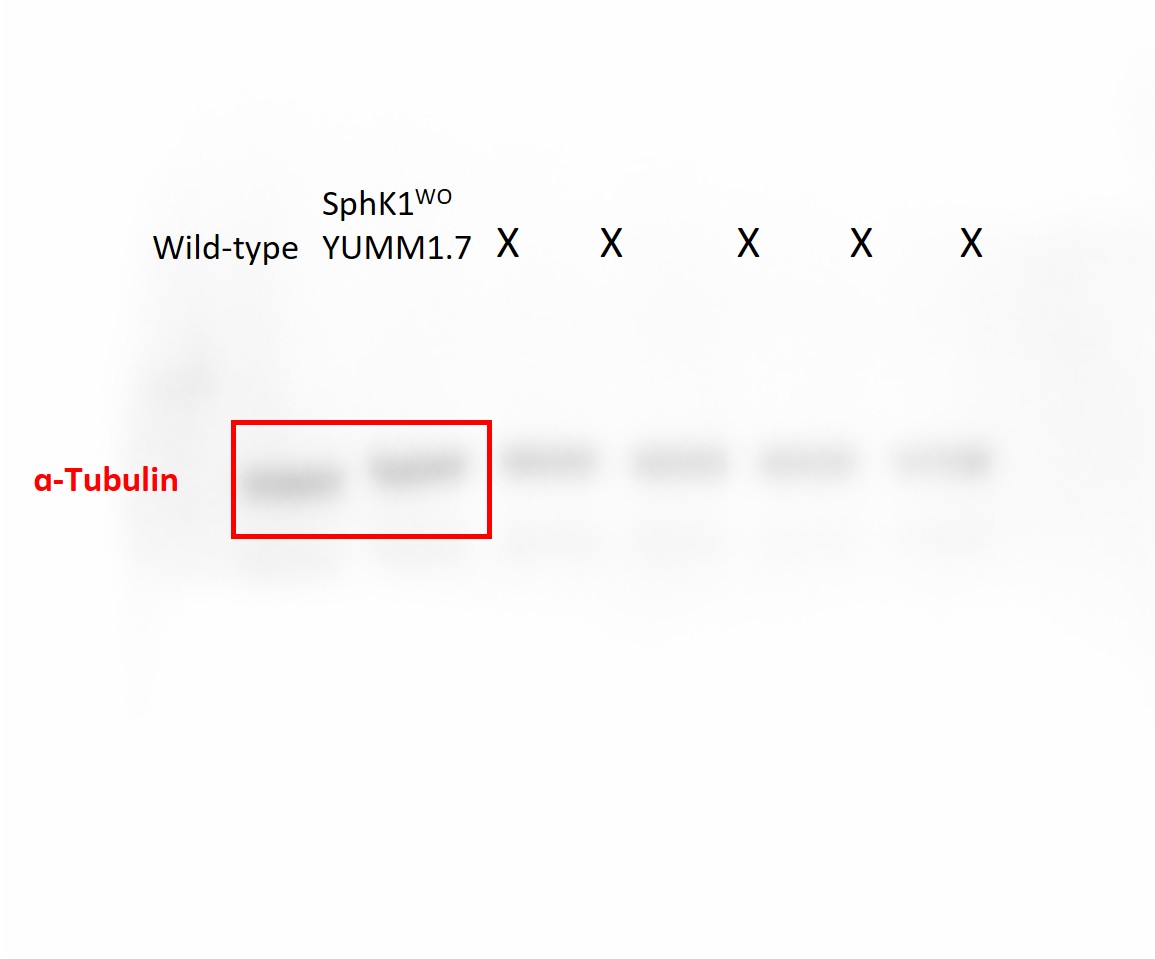

Supplement: Supplementary file 10 — Figure EV3 Source Data [file 44319_2026_734_MOESM10_ESM.zip › EV3/3A/a-tubulin.jpg]
