# Supplementary material for: S1P-S1PR1 signaling impairs CD8+ T cell metabolism and effector function in tumors
Source: EMBO Rep. 2026 Mar 19;27(8):2000–28. doi: 10.1038/s44319-026-00734-3 (PMC13121723; doi:10.1038/s44319-026-00734-3)
Supplement: Supplementary file 13 — Expanded View Figures [file 44319_2026_734_MOESM13_ESM.pdf]

Expanded View Figures

Figure EV1. S1P-S1PR1 signaling modulates exhaustion profiles of $CD8^+$ T cells in TME and chronic stimulation models.

(**A–C**) Gating strategy used for flow cytometry analysis of heterogeneous exhaustion states of $CD8^+$ T cells within the tumor microenvironment (TME) of (A) EL4 thymoma ($n = 4$), (**B**) B16F10 melanoma ($n = 5$), (**C**) YUMM1.7 melanoma ($n = 7$), distinguished by the differential expression of PD1 and Tim3. (**D–H**) Human $CD8^+$ T cells isolated from healthy donor PBMCs were activated for 3 days and then subjected to either continuous TCR stimulation or cultured (without TCR stimulation) with IL-2 for 15 days. Cells were analyzed for (**D**) exhaustion-associated surface markers, (**E**) intracellular production of IFNγ and TNFα, (**F**) intracellular expression of transcription factor TCF1, (**G**) intracellular expression of cell proliferation marker Ki67, and (**H**) cell death using Annexin V and 7AAD. The adjacent bar diagram represents cumulative data from $n = 4$ biological replicates. (**I–M**) $CD8^+$ T cells isolated from the spleen of wild-type B6 mice were activated for 2 days and then subjected to either continuous TCR stimulation or cultured (without TCR stimulation) with IL-2 culture for 9 days. Cells were assessed for (**I**) exhaustion-associated surface markers, (**J**) intracellular production of IFNγ and TNFα, (**K**) intracellular expression of the transcription factor TCF1, (**L**) intracellular expression of cell proliferation marker Ki67, and (**M**) cell death using Annexin V and 7AAD. The adjacent bar diagram represents cumulative data from $n = 4$ biological replicates. (**N**, **O**) Flow cytometry analysis of S1PR1 expression in acutely versus chronically stimulated human (**N**) and murine (**O**) $CD8^+$ T cells. The adjacent bar diagram represents cumulative data from $n = 4$ biological replicates. (**P**) Flow cytometry analysis of p-STAT3 expression in $CD8^+$ T cells isolated from spleens versus tumor tissues, with adjacent bar graphs representing cumulative results from four biological replicates ($n = 4$). (**Q**) Purified mouse $CD8^+$ T cells activated in the presence or absence of S1P were assessed for flow cytometry-based expression of CD25. The adjacent bar diagram represents cumulative data from three biological replicates ($n = 3$). $*P < 0.05$; $**P < 0.01$; $***P < 0.005$; $****P < 0.0001$; ns, nonsignificant ($P > 0.05$), the error bar represents the standard deviation (SD). P values are derived from unpaired two-tailed Student's t test (**A–P**). Source data are available online for this figure.

 © The Author(s)

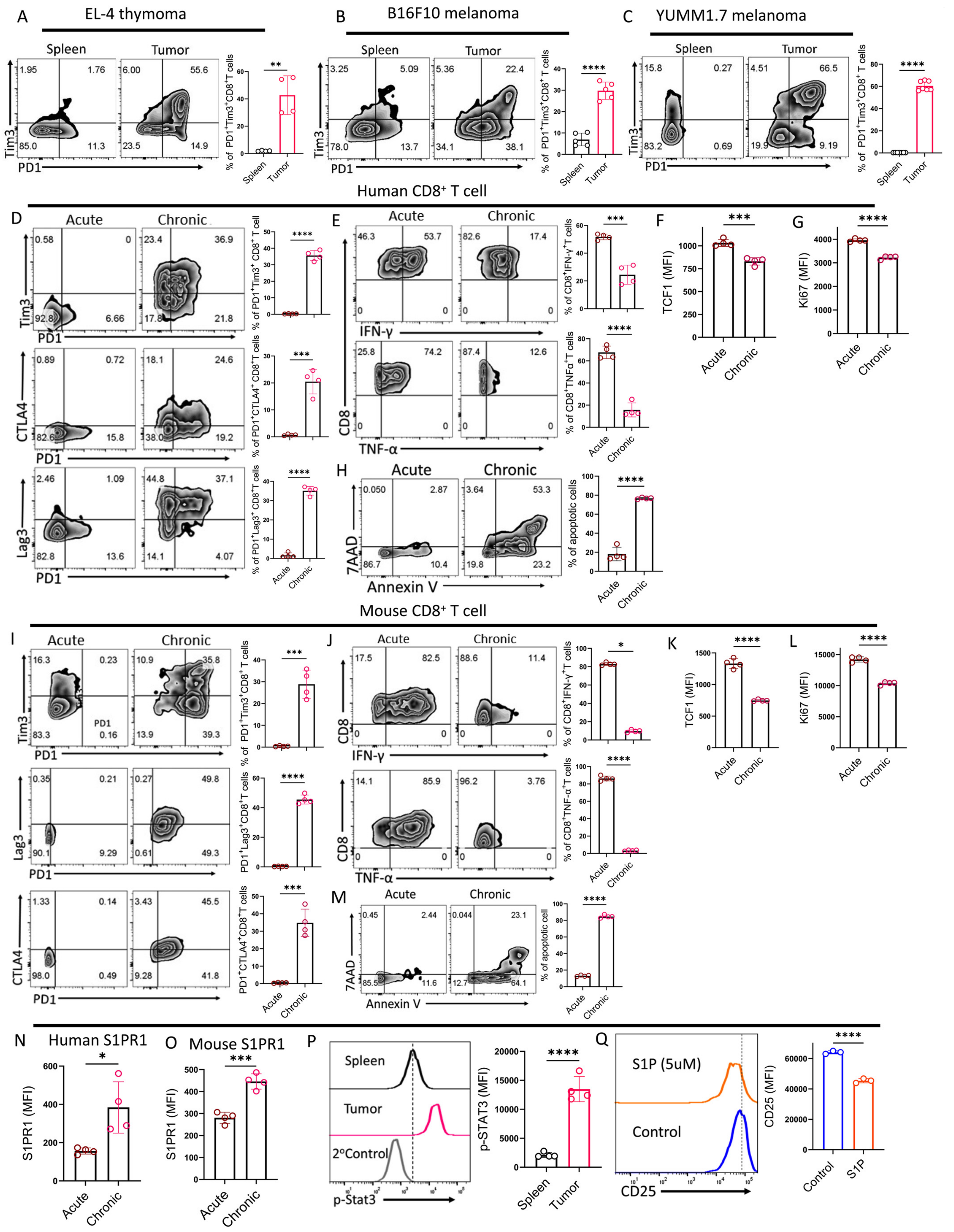
A
EL-4 thymoma
B
B16F10 melanoma
C
YUMM1.7 melanoma
Spleen
Tumor
Human CD8+ T cell
D
Acute
Chronic
E
F
G
H
Mouse CD8+ T cell
I
J
K
L
M
N
Human S1PR1
O
Mouse S1PR1
P
Q
S1P (5uM)
Control
2°Control
p-Stat3
p-STAT3 (MFI)
CD25
CD25 (MFI)
S1PR1 (MFI)
TCF1 (MFI)
Ki67 (MFI)
% of apoptotic cells
Annexin V
7AAD
IFN-γ
TNF-α
CD8
PD1
Tim3
CTLA4
Lag3

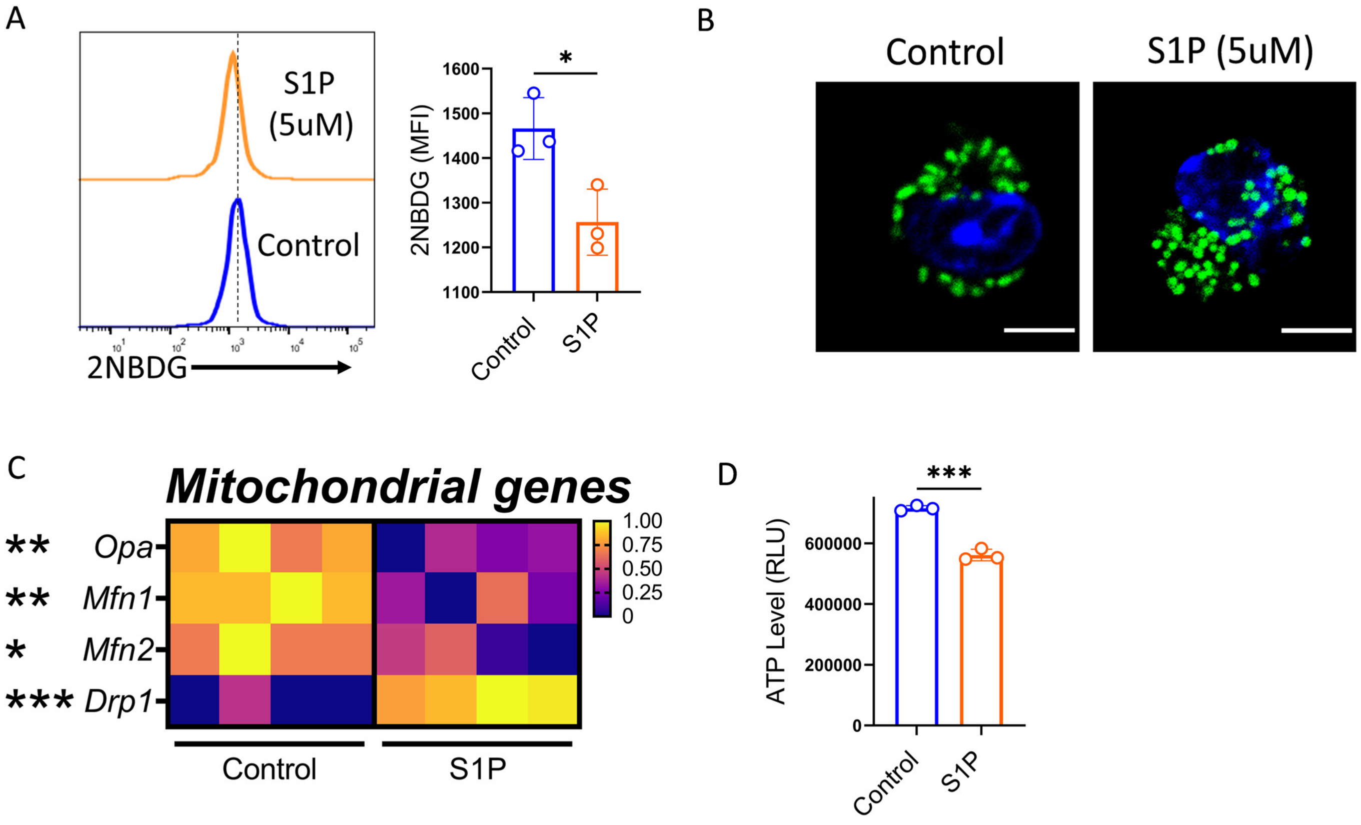

Figure EV2. S1P-S1PR1 signaling modulates metabolic profiles of CD8⁺ T cells.

(**A**) Purified mouse CD8⁺ T cells activated in the presence or absence of S1P were assessed for glucose uptake using 2NBDG ($n = 3$). (**B**) Confocal imaging of activated CD8⁺T cells in the presence and absence of S1P. (**C**) qPCR analysis showing transcript levels of different genes involved in the mitochondrial pathway in respective groups ($n = 4$). (**D**) Graphical representation of the intracellular ATP level in S1P or vehicle-treated CD8⁺ T cells ($n = 3$). *$P < 0.05$; **$P < 0.01$; ***$P < 0.005$; ****$P < 0.0001$; ns, nonsignificant ($P > 0.05$), the error bar represents the standard deviation (SD). P values are derived from unpaired two-tailed Student's t test (**A**, **D**), and two-way ANOVA test (**C**). Source data are available online for this figure.

 © The Author(s)

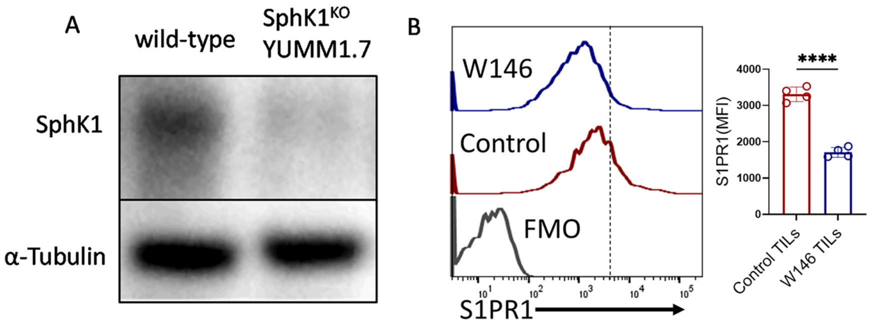

Figure EV3. SphK1 deletion in tumor cells and local S1PR1 inhibition reduce S1PR1 level in tumor-infiltrating T cells.

(**A**) Western blot analysis showing expression of SphK1 in wild-type and SphK1^{KO} YUMM1.7 cells ($n = 3$). (**B**) Flow cytometry analysis of the surface expression of S1PR1 on intratumoral CD8^{+} T cells following administration of S1PR1 antagonist W146 or vehicle control in YUMM1.7 tumor-bearing mice ($n = 4$). *$P < 0.05$; **$P < 0.01$; ***$P < 0.005$; ****$P < 0.0001$; ns, nonsignificant ($P > 0.05$), the error bar represents the standard deviation (SD). P values are derived from unpaired two-tailed Student's t test (**B**). Source data are available online for this figure.

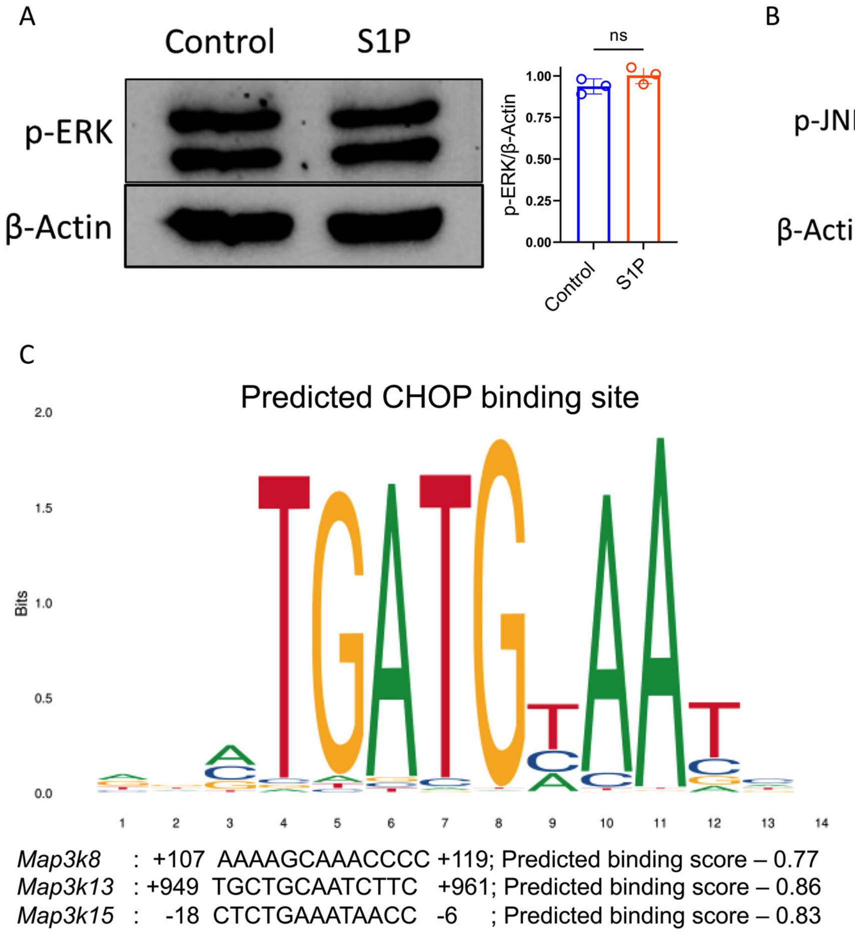

Figure EV4. Evaluation of different MAPK signaling by S1P-S1PR1 signaling.

(**A**, **B**) Western blot analysis showing the expression of (A) p-ERK and (**B**) p-JNK in in-vitro activated CD8$^+$ T cells either in the presence or absence of S1P. The adjacent bar graph depicts normalized densitometric data from three biological replicates ($n = 3$). (**C**) Predicted binding sites of CHOP on the *Map3k8*, *Map3k13*, and *Map3k15* promoters generated from JASPER Software. *$P < 0.05$; **$P < 0.01$; ***$P < 0.005$; ****$P < 0.0001$ ns, nonsignificant ($P > 0.05$), the error bar represents the standard deviation (SD). *P* values are derived from unpaired two-tailed Student's *t* test (**A**, **B**). Source data are available online for this figure.

 © The Author(s)

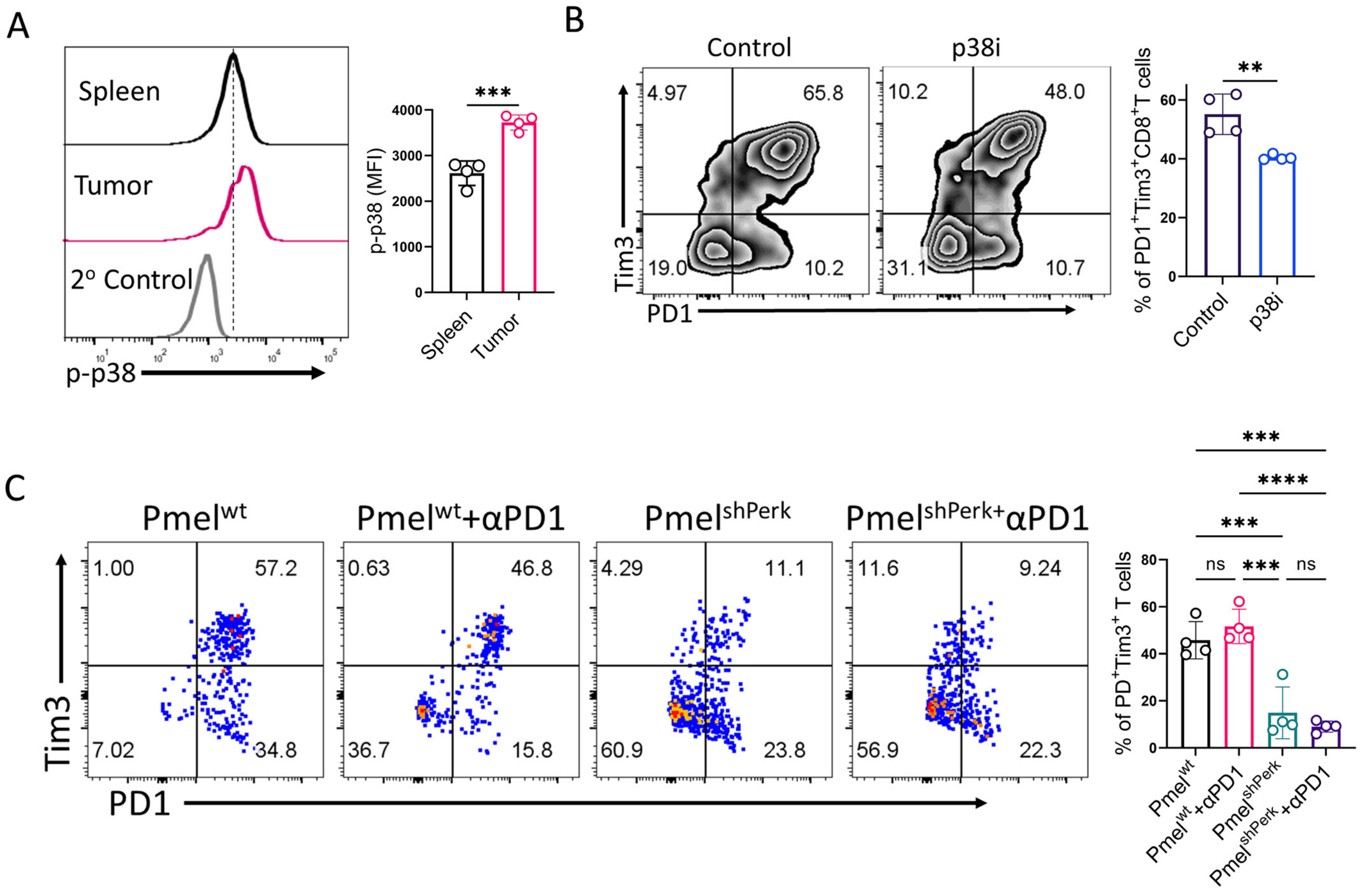

Figure EV5. Evaluation of intratumoral CD8⁺ T cells.

(**A**) CD8⁺ T cells isolated from either the tumor site or spleen of C57BL/6 mice ($n = 4$) bearing YUMM1.7 melanoma were assessed for p-p38 expression. The adjacent bar plot summarizes pooled data from four tumor-bearing mice. (**B**) Intratumoral CD8⁺ T cells from C57BL/6 mice ($n = 4$/group) with subcutaneous YUMM1.7 melanoma, treated with vehicle control or p38i, were evaluated for the frequency of terminally exhausted CD8⁺ T cells (PD1⁺Tim3⁺). The adjacent bar plot summarizes pooled data from four mice per group. (**C**) Adoptively transferred Pmel-1 T cells transduced with either control shRNA or shRNA targeting PERK, isolated from tumors of C57BL/6 mice ($n = 4$/group) bearing subcutaneous B16-F10 melanoma and treated with or without anti-PD1 antibody, were evaluated for the frequency of terminally exhausted CD8⁺ T cells (PD1⁺Tim3⁺). The adjacent bar plot summarizes pooled data from four mice per group. *$P < 0.05$; **$P < 0.01$; ***$P < 0.005$; ****$P < 0.0001$ ns, nonsignificant ($P > 0.05$). The error bar represents the standard deviation (SD). P values are derived from unpaired two-tailed Student's t test (**A**, **B**) and one-way ANOVA. Source data are available online for this figure.
